# Supplementary material for: A set of multi-entry identification keys to African frugivorous flies (Diptera, Tephritidae)
Source: Zookeys. 2014 Jul 24;(428):97–108. doi: 10.3897/zookeys.428.7366 (PMC4143993; doi:10.3897/zookeys.428.7366)
Supplement: Supplementary material 6 — Key to Ceratitis [file zookeys-428-097-s006.zip › SF6_ZooKeys_key to Ceratitis/key/SF6_ZooKeys_key to Ceratitis/Media/Html/Ceratitis cuthbertsoni.htm]

Ceratitis (Pardalaspis) cuthbertsoni (Munro) 


***Ceratitis*** **(*Pardalaspis*)
*cuthbertsoni* (Munro)**

*Pardalaspis* *cuthbertsoni* Munro, 1936: 42.

*Pardalaspis* *cuthbertsoni* var. *nigrotertius*
Munro, 1939: 4.

Body
length: 7.94 (7.5-8.5) mm; wing length: 8.0 (7.0-9.0) mm.

Male

Head.
Antennal segments orange to dark orange. Third antennal segment at most twice
as long as second segment. Arista basal part orange, apically darker. Frons
orange to dark red in ground color, lower third silvery as a distinct band;
dispersed short hairs, only slightly darker than ground colour. Ocellar
triangle dark. Face orange-red; with median whitish band. Occiput moderately
swollen below, pale whitish. Chaetotaxy normal for subgenus.

Thorax.
Ground colour of mesonotum brownish (dark grey in var. *nigrotertius*); 
no distinct spots, three poorly defined darker stripes from anterior margin to
dorsocentral setae, darker area between dorsocentral setae and prescutellar
acrostichal setae especially around the latter, these markings not always
distinct and often interrupted. Postpronotum same colour as centre of mesonotum
(in var. *nigrotertius* paler). Chaetotaxy normal for subgenus. Mesonotum
with pale pilosity. Anepisternum with pale pilosity except upper margin with
darker hairs. Two anepisternal bristles.  Scutellum yellow with apical markings
black; basally without dark spots, only faintly darker yellow coloration.

Legs
dark yellow to orange brown; setation typical of subgenus. Front femur with
mainly pale pilosity, posterior row dark

Wings
with brownish bands, yellow markings strongly reduced. Banding, setation and
venation normal for subgenus. Marginal band interrupted at vein R_1_;
discal band joined with marginal band but interrupted in discal cell (in some
specimens interruption is very weak and bands appear continuous); cross-vein
r-m usually before middle of discal cell, sometimes at middle; vein R_1_
usually ending opposite or beyond cross-vein r-m.

Abdomen.
grey-brown, with clearly defined spots. Pattern of spots and setation normal
for subgenus.

Female

As
male except for the following characters: Frons yellow to yellow-orange, lower
margin distinctly paler. Face wholly yellow. Markings on mesonotum less clearly
defined. In var. *nigrotertius* position of R_1_ variable, either
before or beyond cross-vein. Oviscape very long, about twice as long as
abdominal terga 3-6; dark orange to brown.    

(Description
after De Meyer, 1996)
